# Deaths from Falls Among Persons Aged ≥65 Years — United States, 2007–2016

**DOI:** 10.15585/mmwr.mm6718a1

**Published:** 2018-05-11

**Authors:** Elizabeth Burns, Ramakrishna Kakara

**Affiliations:** ^1^Division of Unintentional Injury, National Center for Injury Prevention and Control, CDC; ^2^Oak Ridge Institute for Science and Education (ORISE) fellow.

Deaths from unintentional injuries are the seventh leading cause of death among older adults (*1*), and falls account for the largest percentage of those deaths. Approximately one in four U.S. residents aged ≥65 years (older adults) report falling each year (*2*), and fall-related emergency department visits are estimated at approximately 3 million per year.* In 2016, a total of 29,668 U.S. residents aged ≥65 years died as the result of a fall (age-adjusted rate^†^ = 61.6 per 100,000), compared with 18,334 deaths (47.0) in 2007. To evaluate this increase, CDC produced age-adjusted rates and trends for deaths from falls among persons aged ≥65 years, by selected characteristics (sex, age group, race/ethnicity, and urban/rural status) and state from 2007 to 2016. The rate of deaths from falls increased in the United States by an average of 3.0% per year during 2007–2016, and the rate increased in 30 states and the District of Columbia (DC) during that period. In eight states, the rate of deaths from falls increased for a portion of the study period. The rate increased in almost every demographic category included in the analysis, with the largest increase per year among persons aged ≥85 years. Health care providers should be aware that deaths from falls are increasing nationally among older adults but that falls are preventable. Falls and fall prevention should be discussed during annual wellness visits, when health care providers can assess fall risk, educate patients about falls, and select appropriate interventions.

Mortality data from death certificates filed in 50 states and DC were analyzed to determine the number of deaths from falls among persons aged ≥65 years by selected characteristics, year, and state in which the death occurred. Each certificate identifies demographic data and a single underlying cause of death. Falls were identified using *International Classification of Diseases, Tenth Revision* codes W00–W19. Queries to CDC WONDER^§^ were used to generate the 2007 and 2016 age-specific rates for three age groups (65–74, 75–84, and ≥85 years) and age-adjusted rates by sex, race/ethnicity (non-Hispanic white, non-Hispanic black, American Indian/Alaska Native, Asian/Pacific Islander, or Hispanic), and urban/rural status.^¶^ The years 2007–2016 were selected to produce 10-year age-adjusted trends for the United States, 49 U.S. states,** and DC. Population estimates produced by the U.S. Census with CDC’s National Center for Health Statistics were used to calculate mortality rates. Age-standardized rates were produced using the 2000 U.S. standard population. All rates in this report are age-adjusted and restricted to adults aged ≥65 years.

National and state-specific trends were evaluated using joinpoint software,^††^ which identifies statistically significant changes in a trend using Monte Carlo permutation, then fits them as a series of joined trend segments. An annual percentage change (APC) for each segment, an average APC (AAPC) for the 10 years, and confidence intervals at α = 0.05 were calculated.

The overall rate of older adult deaths from falls increased 31% from 2007 to 2016 (3.0% per year) (Figure 1). Nationwide, 29,668 (61.6 per 100,000) U.S. residents aged ≥65 years died from fall-related causes in 2016. State-specific rates ranged from 24.4 (Alabama) to 142.7 (Wisconsin) (Figure 2) (Supplementary Table; https://stacks.cdc.gov/view/cdc/53652). The largest AAPC in mortality rates from falls (11.0% per year) occurred in Maine, followed by Oklahoma (10.9%) and West Virginia (7.8%). A significant increase in the rate from 2007 to 2016 occurred in 30 states (Arkansas, California, Connecticut, Florida, Idaho, Illinois, Indiana, Iowa, Kansas, Kentucky, Louisiana, Maine, Maryland, Massachusetts, Michigan, Minnesota, Montana, New Jersey, New York, North Carolina, Ohio, Oklahoma, Pennsylvania, Rhode Island, South Carolina, South Dakota, Virginia, Washington, West Virginia, and Wyoming) and DC. No significant change in fall mortality rates occurred in 11 states (Alabama, Delaware, Georgia, Hawaii, Mississippi, Nebraska, New Hampshire, New Mexico, North Dakota, Texas, and Vermont). After an initial increase, rates stabilized in three states (Colorado, Oregon, and Tennessee). Arizona, Nevada, and Wisconsin had initial periods of stability followed by a significant increase in fall death rates. The death rate from falls decreased in Missouri during 2007–2012, followed by an increase during 2012–2016, and increased in Utah during 2007–2012, followed by a decrease during 2012–2016.

**FIGURE 1:**
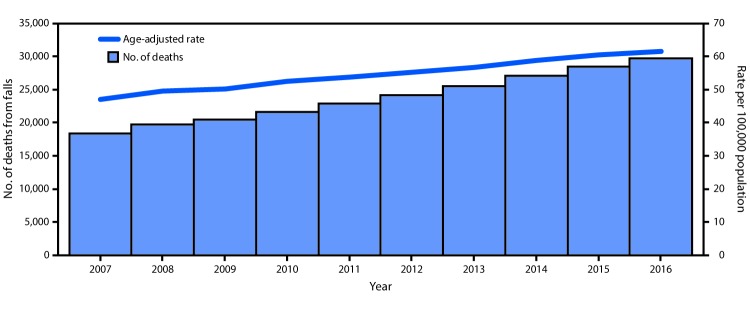
Number of deaths from falls and age-adjusted rates* among adults aged ≥65 years — United States, 2007–2016 * Age-adjusted death rates were calculated by applying age-specific death rates to the 2000 U.S standard population age distribution.

**FIGURE 2:**
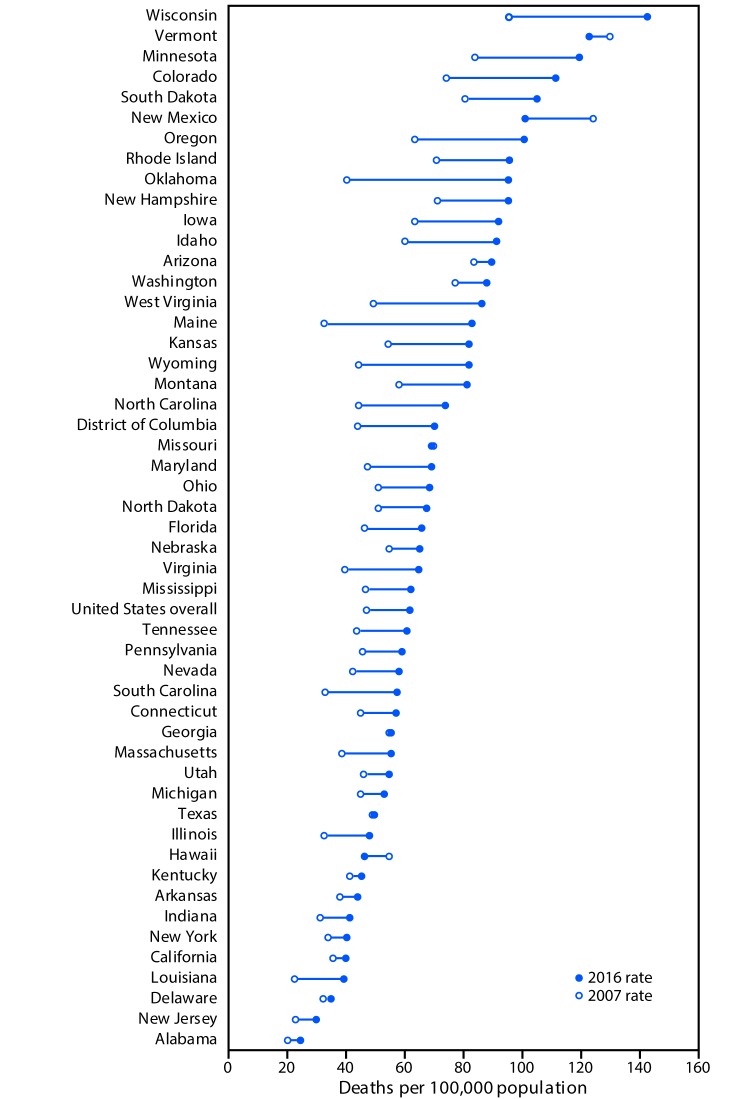
Age-adjusted rate* of deaths from falls**^†^** among persons aged ≥65 years, by state and overall — United States, 2007 and 2016^§^ **Source:** CDC. National Vital Statistics System, Mortality. CDC WONDER. https://wonder.cdc.gov/. * Rates shown are the number of deaths per 100,000 population. Age-adjusted death rates were calculated by applying age-specific death rates to the 2000 U.S standard population age distribution. ^†^ Deaths from falls were identified using *International Classification of Diseases, Tenth Revision* (ICD–10) underlying cause-of-death codes W00–W19. ^§^ Joinpoint regression examining changes in trends indicated that, from 2007 to 2016, the District of Columbia and 30 states had significant increases in the rate of deaths from falling (Arkansas, California, Connecticut, Florida, Idaho, Illinois, Indiana, Iowa, Kansas, Kentucky, Louisiana, Maine, Maryland, Massachusetts, Michigan, Minnesota, Montana, New Jersey, New York, North Carolina, Ohio, Oklahoma, Pennsylvania, Rhode Island, South Carolina, South Dakota, Virginia, Washington, West Virginia, and Wyoming). Colorado, Oregon, and Tennessee had initial increases, followed by stable rates during this period. Arizona, Nevada, and Wisconsin had an initial period of stability followed by a significant increase. In Missouri, there was a decrease from 2007 to 2012, followed by an increase from 2012 to 2016. In Utah there was an increase from 2007 to 2012 followed by a decrease to 2016. Eleven states had nonsignificant trends during this period (Alabama, Delaware, Georgia, Hawaii, Mississippi, Nebraska, New Hampshire, New Mexico, North Dakota, Texas, and Vermont). Alaska did not have enough data to examine trends.

In 2016, death rates from falls were higher among adults aged ≥85 years (257.9), men (72.3), and whites (68.7) than among corresponding groups (Table). From 2007 to 2016, rates increased among all demographic subgroups except American Indians/Alaska Natives. The annual rate increase was larger among adults aged ≥85 years (3.9% per year) than among those aged 65–74 years (1.8%) and 75–84 years (2.3%).

**TABLE:** Number and age-adjusted rates* for deaths from falls and annual percentage changes^†^ among persons aged ≥65 years, by selected characteristics — United States, 2007–2016

Characteristic	2007		2016		2007–2016
	No. of deaths	Deaths per 100,000 (95% CI)	No. of deaths	Deaths per 100,000 (95% CI)	APC (95% CI)
**Total**	**18,334**	**47.0 (46.4–47.7)**	**29,668**	**61.6 (60.9–62.3)**	**3.0 (2.8–3.2)**
**Sex**					
Men	8,408	57.9 (56.7–59.2)	13,721	72.3 (71.1–73.5)	2.4 (2.1–2.7)
Women	9,926	40.2 (39.4–41.0)	15,947	54.0 (53.1–54.8)	3.8 (3.2–4.4)
**Age group (yrs)**					
65–74	2,594	13.2 (12.7–13.7)	4,479	15.6 (15.2–16.1)	1.8 (1.3–2.3)
75–85	6,552	50.1 (48.9–51.3)	8,735	61.4 (60.1–62.7)	2.3 (1.8–2.7)
≥85	9,188	182.3 (178.6–186.0)	16,454	257.9 (253.9–261.8)	3.9 (3.7–4.0)
**Race/Ethnicity^§^**					
White, non-Hispanic	16,609	50.7 (49.9–51.4)	26,370	68.7 (67.8–69.5)	3.4 (3.2–3.6)
Black, non-Hispanic	595	19.9 (18.3–21.5)	1,089	27.1 (25.5–28.7)	3.2 (2.1–4.4)
American Indian/Alaska Native	74	47.3 (36.9–59.8)	111	47.0 (38.1–55.9)	−1.5 (−3.6–0.6)
Asian/Pacific Islander	343	31.1 (27.8–34.4)	738	36.7 (34.0 –- 39.4)	1.5 (0.7–2.4)
Hispanic	681	32.4 (29.9–34.9)	1,296	35.7 (33.8–37.7)	1.2 (0.2–2.2)
**Urban/Rural status^¶^**					
Large central metro	5,008	47.4 (46.1–48.7)	7,442	57.0 (55.7–58.3)	2.2 (1.9–2.4)
Large fringe metro	3,990	44.0 (42.7–45.4)	7,000	59.9 (58.5–61.3)	3.4 (2.6–4.2)
Medium metro	4,008	48.3 (46.8–49.8)	6,879	66.1 (64.5–67.7)	3.3 (2.9–3.7)
Small metro	1,918	49.3 (47.1–51.5)	3,186	66.4 (64.1–68.7)	3.3 (2.5–4.0)
Micropolitan (non-metro)	1,976	49.6 (47.4–51.8)	2,970	64.2 (61.9–66.6)	2.8 (2.4–3.3)
Non-core (non-metro)	1,434	44.9 (42.6–47.2)	2,191	60.9 (58.3–63.5)	3.3 (3.0–3.7)

**Source:** CDC, National Vital Statistics System, Mortality. CDC WONDER. https://wonder.cdc.gov/.

**Abbreviations**: APC = annual percentage change; CI = confidence interval.

* Rates standardized to the 2000 U.S. population with age groups 65–74, 75–84, and ≥85 years.

^†^ The annual percentage change was also the average annual percentage change for the years 2007–2016 because no significant change in trend was identified during this period using joinpoint regression.

^§^ Persons in the four racial categories were all non-Hispanic. Hispanic persons might be of any race.

^¶^ Status follows the 2013 Urban-Rural Classification Scheme for Counties of CDC’s National Center for Health Statistics.

## Discussion

Approximately 30,000 adults aged ≥65 years died as the result of a fall in 2016, and state-specific rates for deaths from falls ranged from 24.4 per 100,000 in Alabama to 142.7 in Wisconsin. The rate of deaths from falls among older adults increased steadily from 2007 to 2016 in 30 states and DC. The 31% increase in the national rate of deaths from falls from 2007 to 2016 is consistent with findings from a 2010 study that estimated a 42% increase from 2000 to 2006 (*3*).

The differences in rates among states might have resulted, in part, from differences in the racial composition or general health of the states’ residents. For example, in 2016, the rate of deaths from falls was higher among older white adults than among other racial/ethnic groups. Thus, the higher rate in Wisconsin, compared with that in Alabama, might be partially attributable to a higher proportion of white older adults in Wisconsin than in Alabama.^§§^ Differential coding practices for external causes of injury on the death certificate might also contribute to variation in both the rate and APC (*4*,*5*). In addition, some states require a medical examiner to complete a death certificate, whereas others employ coroners; a 2012 study of national trends and coding patterns in fall-related mortality among the elderly found that coroners recorded 14% fewer deaths from falls than did medical examiners (*5*).

In 2016, there was a higher rate of fatal falls among older men, in contrast to the rate of nonfatal falls, which is higher among older women (*2*). This might have resulted from differences in the circumstance of a fall (e.g., from a ladder or while drinking) (*6*,*7*), leading to more serious injuries, including head trauma, or higher rates of postfall complications in men (*7*). The higher rates of deaths from falls among older age groups is consistent with advancing age being an independent risk factor for falls as well as being associated with other risk factors such as 1) reduced activity; 2) chronic conditions, including arthritis, neurologic disease, and incontinence; 3) increased use of prescription medications, which might act synergistically on the central nervous system; and 4) age-related changes in gait and balance (*8*).

The population of older adults in the United States is increasing; adults aged ≥85 years are the fastest-growing age group among U.S. residents and will reach approximately 8.9 million in 2030 (*9*). Although the rate of deaths from falls is increasing among all persons aged ≥65 years, it is increasing fastest among those aged ≥85 years (3.9% per year). Nationally, the rate of deaths from falls might be increasing because of longer survival after the onset of common diseases such as heart disease, cancer, and stroke (*6*). If the current rate remains stable, an estimated 43,000 U.S. residents aged ≥65 years will die because of a fall in 2030, and if the rate continues to increase, 59,000 fall-related deaths could result.

The findings in this report are subject to at least five limitations. First, changes in coding of cause of death might have occurred during the study period, which might contribute to the increased rate of deaths from falls. Second, information about race and Hispanic ethnicity is generally reported by the funeral director and might be based on observation, which could lead to an underestimation of deaths among Hispanics, Asians/Pacific Islanders, and American Indians/Alaska Natives.^¶¶^ Third, the age-adjusted rates were based on information from the U.S. Census, which reports as a limitation that it might undercount persons aged ≥65 years; this could result in an overestimation of death rates. Fourth, misclassifications of deaths might have produced overestimates or underestimates of deaths from falls. Finally, standard age-adjusted populations might not fully adjust populations at older age groups (e.g., ≥85 years) and could explain differences between subgroups and states.

As the population of persons aged ≥65 years in the United States, increases, the rising number of deaths from falls in this age group can be addressed by screening for fall risk and intervening to address modifiable risk factors such as polypharmacy or gait, strength, and balance issues. Interventions that target multiple risk factors can reduce the rate of falls (*10*) and can be initiated during annual wellness visits.*** Initiatives such as CDC’s STEADI (Stopping Elderly Accidents, Deaths, and Injuries),^†††^ can assist health care providers in assessing fall risk, educating patients, and selecting interventions.

**Summary**

**What is already known about this topic?**

Falls are the leading cause of injury-related deaths among persons aged ≥65 years, and the age-adjusted rate of deaths from falls is increasing.

**What is added by this report?**

The rate of deaths from falls among persons aged ≥65 years increased 31% from 2007 to 2016, increasing in 30 states and the District of Columbia, and among men and women. Among states in 2016, rates ranged from 24.4 per 100,000 (Alabama) to 142.7 (Wisconsin). The fastest-growing rate was among persons aged ≥85 years (3.9% per year).

**What are the implications for public health practice?**

As the U.S. population aged ≥65 years increases, health care providers can address the rising number of deaths from falls in this age group by asking about fall occurrences, assessing gait and balance, reviewing medications, and prescribing interventions such as strength and balance exercises or physical therapy.

## Footnotes

* https://www.cdc.gov/injury/wisqars.

† All rates in this report are age-adjusted and restricted to adults aged ≥65 years.

§ https://wonder.cdc.gov/.

¶ 2013 NCHS Urban-Rural Classification Scheme for Counties. https://www.cdc.gov/nchs/data/series/sr_02/sr02_166.pdf.

** Alaska did not have enough data to examine trends.

†† For all analyses, a p-value of <0.05 was considered to be statistically significant. https://surveillance.cancer.gov/joinpoint/.

§§
https://factfinder.census.gov/faces/tableservices/jsf/pages/productview.xhtml?pid=ACS_16_5YR_DP05&src=pt .

¶¶ https://wonder.cdc.gov/wonder/help/cmf.html#.

*** https://www.medicareinteractive.org/get-answers/medicare-covered-services/preventive-care-services/annual-wellness-visit.

††† https://www.cdc.gov/steadi/.
